# The Culture of Libyan Society Regarding COVID-19

**DOI:** 10.1017/dmp.2020.502

**Published:** 2020-12-22

**Authors:** Abdalla Mohamed Bashir

**Affiliations:** Research and Consulting Center (RCC), University of Benghazi, Benghazi, Libya

I am writing to support the claims made by some Libyan authors who conducted studies about affecting the spread of the coronavirus disease (COVID-19) pandemic in Libya.^1–3^ One of the authors states that, at the beginning of the spread of the COVID-19 pandemic, the Libyan Government had implemented precaution measures in controlling the spread of the virus.^1^ Others declare that there is a lack of awareness and willingness among health care workers in the face of COVID-19.^2,3^ The authors of these studies observed that the majority of health care workers are unaware of dealing with the COVID-19 pandemic.

Further, authors have attempted to explore the various social, psychological, economic, and health aspects affected during the COVID-19 pandemic. According to this study, the social aspect was the lowest effect by the spread of COVID-19, while other aspects were ranged between middle and high effects, respectively. Thus, it is very important for the Libyan Government and the Libyan National Centre for Disease Control to take the results of the previous studies into account to limit the spread of COVID-19.

In reviewing the above studies, the researcher agrees with the Libyan authors who declare that Libyans did not seriously follow the preventive measures, for example, social distancing particularly in social occasions. The researcher also agrees with them that most Libyans are concerned about the absence of health care in the hospitals if they see a doctor due to the emergence of COVID-19 symptoms.

However, the previous authors ignored some of observations, for example, the researcher noticed that some Libyans make fun of those who wear face masks and those who adhere to social distancing, especially in the public places. This is because they do not take this matter very seriously, while others are of the opinion that there is no COVID-19 at all in Libya. This is because they do not trust the Libyan National Centre for Disease Control. In addition, the Libyan Government did not provide medical materials, especially in public places. Not only this, but also it did not take into account those who work for themselves. This, in turn, leads to an increased number of cases affected with COVID-19 in this country. This letter is only a simple snapshot of the culture and behavior of some people in the Libyan community during the COVID-19 pandemic. It explained how some Libyans are aware of and coping with the spread of the COVID-19 pandemic. Therefore, educating people about the danger of the spread of COVID-19 is extremely important.
